# Validity and accuracy of the Adult Attention‐Deficit/Hyperactivity Disorder (ADHD) Self‐Report Scale (ASRS) and the Wender Utah Rating Scale (WURS) symptom checklists in discriminating between adults with and without ADHD

**DOI:** 10.1002/brb3.1605

**Published:** 2020-04-13

**Authors:** Erlend Joramo Brevik, Astri J. Lundervold, Jan Haavik, Maj‐Britt Posserud

**Affiliations:** ^1^ Division of Psychiatry Haukeland University Hospital Bergen Norway; ^2^ Department of Biomedicine K.G. Jebsen Centre for Neuropsychiatric Disorders University of Bergen Bergen Norway; ^3^ Department of Biological and Medical Psychology University of Bergen Bergen Norway; ^4^ Department of Clinical Medicine University of Bergen Bergen Norway

**Keywords:** adult, attention deficit disorder with hyperactivity, checklist, psychometrics

## Abstract

**Objective:**

To validate the Adult ADHD Self‐Report Scale (ASRS) and the Wender Utah Rating Scale (WURS) in a well‐characterized sample of adult attention‐deficit/hyperactivity disorder (ADHD) patients and population controls.

**Methods:**

Both the ASRS and the WURS were administered to clinically diagnosed adult ADHD patients (*n* = 646) and to population controls (*n* = 908). We performed principal component analyses (PCA) and calculated receiver operating curves (ROC) including area under the curve (AUC) for the full WURS and ASRS, as well as for the PCA generated factors and the ASRS short screener.

**Results:**

We found an AUC of 0.956 (95% CI: 0.946–0.965) for the WURS, and 0.904 (95% CI: 0.888–0.921) for the ASRS. The ASRS short screener had an AUC of 0.903 (95%CI: 0.886–0.920). Combining the two full scales gave an AUC of 0.964 (95% CI: 0.955–0.973). We replicated the two‐factor structure of the ASRS and found a three‐factor model for the WURS.

**Conclusion:**

The WURS and the ASRS both have high diagnostic accuracy. The short ASRS screener performed equally well as the full ASRS, whereas the WURS had the best discriminatory properties. The increased diagnostic accuracy may be due to the wider symptom range of the WURS and/or the retrospective childhood frame of symptoms.

## Graphical abstract

To validate the adult ADHD Self‐Report Scale (ASRS) and the Wender Utah Rating Scale (WURS) in a well‐validated sample of adult attention‐deficit/hyperactivity disorder (ADHD) patients and population controls. Both the ASRS and the WURS was administered to clinically diagnosed adult ADHD patients (*n* = 646) and population controls (*n* = 908). The short ASRS screener performed equally well as the full ASRS, whereas the WURS had the best discriminatory properties.

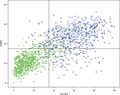

## Significant Outcomes

- The Norwegian Wender Utah Rating Scale (WURS) and Adult ADHD Self‐Report Scale (ASRS) were validated, both demonstrating excellent screening properties.
- Retrospective childhood symptoms of aggressiveness and social problems are highly predictive of an adult diagnosis of attention‐deficit hyperactivity disorder.
- Our results support that emotional regulation problems constitute a large part of ADHD symptomatology in childhood.

## Limitations

- The use of retrospective self‐report measures might be affected by memory biases and lack of recall.
- The use of self‐report measures for present ADHD symptoms may be biased by the current health and life situation of the informant.
- This study was based on a sample diagnosed with ADHD as adults, thus it is uncertain whether the patients included would have obtained a childhood diagnosis of ADHD.

## 1 INTRODUCTION

Adult attention‐deficit hyperactivity disorder (ADHD) is a persistent neurodevelopmental disorder with childhood onset, characterized by inattention, hyperactivity, and impulsivity (American Psychiatric Association, 2013). ADHD has a prevalence of about 5% in childhood (Polanczyk, de Lima, Horta, Biederman, & Rohde, 2007), with about half persisting into adulthood (Faraone et al., 2015). As contextual demands continue to increase in number, scope and complexity with age, coupled with decreased support systems, ADHD may first be recognized and diagnosed in adults (Turgay et al., 2012). Fayyad et al. (2017) found an overall prevalence of 2.8% of DSM‐IV adult ADHD across a range of nations, spanning from 1.4% in lower income countries to 3.6% in higher income countries. Adult ADHD is associated with for example lower educational achievement and increased rates of incarcerations, unemployment and illicit drug use (Faraone et al., 2015). Clinical assessment based on the Diagnostic and Statistical Manual of Mental Disorders (DSM) criteria is the gold standard for the diagnosis (Haavik, Halmoy, Lundervold, & Fasmer, 2010), but short screeners or symptom rating scales provide a quick and easy way of obtaining standardized information to select patients for further examination.

It is important to establish a history of childhood ADHD symptoms, as the pharmacological treatment of ADHD involves regulated substances and as several other disorders that appear in adulthood may display ADHD symptoms (e.g., affective disorders, substance use disorders, and sleep disorders; Haavik et al., 2010). To add to the complexity, these disorders may often also be comorbid with ADHD. The Wender Utah Rating Scale (WURS) was developed to retrospectively evaluate the presence and severity of childhood symptoms of ADHD in adult patients (Ward, Wender, & Reimherr, 1993). The WURS is based on the Utah criteria (Wender, 1995), requiring a childhood history of ADHD including both inattentive and hyperactive symptoms, with one of the following additional symptoms: behavior problems in school, impulsivity, over‐excitability and temper outbursts. The Utah criteria also require an adult history of persistent attention problems and motor hyperactivity with at least two of the following symptom domains: emotional lability, hot temper, stress intolerance, disorganization and impulsivity (Ward et al., 1993). The original 61‐item questionnaire was subsequently reduced to the 25 items that best distinguished an ADHD sample from control samples (i.e., healthy controls and depressed patients). Most of the final 25 items are thus not directly tapping into the core ADHD symptoms, but were chosen for their discriminative ability. A recent study has found that emotional lability measured by the WURS may be one of the best childhood predictors of adult ADHD (Gisbert et al., 2018). A WURS‐25 score of at least 36 identified 96% of adults with ADHD and 96% of healthy controls (Ward et al., 1993). A cutoff of 46 or higher correctly identified 86% of adults with ADHD, 99% of “normal” controls, and 81% of a comparison sample with depression. Several authors have reported a 3‐factor structure of the WURS under somewhat different names. McCann, Scheele, Ward, and Roy‐Byrne (2000) named the factors *Dysthymia*, *Oppositional/Defiant Behavior*, and *School Problems* while Caci, Bouchez, and Baylé (2010) named the factors *Impulsivity/Temper*, *Inattentiveness*, and *Mood/Self‐esteem*. Stanton and Watson (2016) recently reported factors *Aggression*, *Internalizing Distress*, and *Academic Difficulties* of the WURS in a community sample.

Current symptoms of inattention and/or hyperactivity and impulsivity are also essential for the diagnosis of ADHD to be made in adulthood. The Adult ADHD Self‐Report Scale (ASRS) is the official screening instrument of the World Health Organization (WHO; Kessler et al., 2005), and includes the 18 items ADHD symptoms of the DSM. It is one of the most commonly used screening instruments of current ADHD symptoms in adults. The authors/creators of the ASRS tested several variants of administering the 18 DSM symptoms of ADHD, and concluded that a 6‐item version was best suited as general population screen (Kessler et al., 2005, 2007). The authors based their conclusion on blind clinical ratings of DSM‐IV adult ADHD in a sample of merely 154 respondents from the US National Comorbidity Survey Replication (NCS‐R), oversampling those who reported childhood ADHD and adult persistence (Kessler et al., 2005). Recently, the same group (Ustun et al., 2017) created an updated 6‐item screen of the ASRS replacing two of the 6 items with items on executive functioning (i.e., not part of the ADHD defining symptoms). They found this to have good psychometric properties as a general population screener. However, another small nonclinical study comparing the short screener to the full 18 items version found the lengthy version to have better psychometric properties (Zohar & Konfortes, 2010). The authors pointed out the need for a direct assessment of the utility of the ASRS in clinical samples, as there is a lack of studies examining the screening properties of the whole ASRS in an adequately large sample of adults with a clinically confirmed ADHD diagnosis and population controls. The ASRS and the 25‐item WURS have been translated into several languages, including Norwegian. Validation studies of multiple versions have shown similar psychometric properties to those reported for the original English versions (Caci et al., 2010; Kessler et al., 2005, 2007; McCann et al., 2000; Stanton & Watson, 2016; Ustun et al., 2017; Zohar & Konfortes, 2010). The Norwegian versions were translated to Norwegian and back‐translated according to commonly accepted protocols. Although these versions have been widely used, we are not aware of official validation studies.

The aims of the present study were threefold: first, to establish the construct and content validity of the Norwegian translations of the WURS and the ASRS using principal component analysis; second, to examine the psychometric properties of the WURS and the ASRS in a large clinically diagnosed adult ADHD patient sample and population controls; third, to compare the utility of these instruments to aid the clinical ADHD diagnosis.

## 2 METHOD

### 2.1 Participants

The participants were recruited as part of the “ADHD in Norwegian Adults” project launched in 2004 with the aim to improve knowledge about ADHD in adults concerning etiology, diagnosis, and treatment. The ADHD sample constitutes a well‐validated group, mainly recruited from a national registry of adults diagnosed in Norway from 1997 to May 2005. As part of a national quality improvement project, all diagnoses of adult ADHD during 1997–2005 had to be evaluated and approved by one out of three expert committees (situated in Oslo, Trondheim and Bergen). When this protocol was terminated in 2005, the same diagnostic protocol was continued, but without the mandatory extra approval. Experienced clinical psychologists and psychiatrists made the diagnostic assessment in routine practice in outpatient clinics, according to the 10th revision of the International Statistical Classification of Diseases and Related Health Problems (ICD‐10; WHO, 1992), with allowances for the subtypes described in the DSM‐IV‐TR (American Psychiatric Association, 2000). Patients were included in the registry regardless of the final decision to administer stimulants as part of their treatment. Comorbidities were assessed and allowed as comorbidities are found to be highly prevalent among patients with ADHD, with mood and anxiety disorders, substance use disorders, and personality disorders being the most frequent, increasing the ecological validity of the final sample (Faraone et al., 2015; Haavik et al., 2010; Halmoy, Fasmer, Gillberg, & Haavik, 2009; Halmoy et al., 2010; Katzman, Bilkey, Chokka, Fallu, & Klassen, 2017). The control sample (18–40 years old at the time of recruitment) was randomly selected from the Medical Birth Registry of Norway (MBRN). All participants provided signed informed consent. The study was approved by the Norwegian Regional Committee for Medical and Health Research Ethics, REC West [IRB #3 (FWA00009490, IRB00001872)].

### 2.2 Instruments

#### 2.2.1 The Wender Utah Rating Scale

The 25‐item version of the Wender Utah Rating Scale (WURS; Ward et al., 1993) assesses childhood symptoms by asking the participants to retrospectively recall the frequency and severity of ADHD symptoms and related problems experienced in childhood. Participants responded to these items on a Likert‐type 5‐point scale according to the following response categories: “not at all/very slightly” (0), “mildly” (1), “moderately” (2), quite a bit” (3), or “very much” (4), giving a possible range of 0–100 points.

#### 2.2.2 The Adult ADHD Self‐Report Scale

The Adult ADHD Self‐Report Scale (ASRS) is a brief screening instrument to identify current ADHD symptoms (Kessler et al., 2005). The scale was developed by the World Health Organization (WHO, 1992) and the Work Group on Adult ADHD (Kessler et al., 2005). The scale contains the 18 symptoms of inattention, hyperactivity, and impulsivity defining ADHD according to the DSM‐IV‐TR and DSM‐5 (American Psychiatric Association, 2000, 2013). The severity of the symptoms are reported on a 5‐point Likert‐type scale (0–4 = never, rarely, sometimes, often, to very often), with a total range of 0–72. The total ASRS score has shown good reliability and validity in both clinical and population samples (Adler et al., 2006; Glind et al., 2013).

### 2.3 Statistics and analytic plan

A Principal Component Analysis (PCA) with Varimax rotation was run to establish how the items of the WURS and the ASRS contributed to given components, selecting components with Eigenvalues above one (we henceforth refer to components as factors; Field, 2013). We calculated receiver operating curves (ROC) including area under the curve (AUC) for the full WURS and ASRS, as well as for the PCA generated factors.

The likelihood ratios for positive tests (LH+) and negative tests (LH−) and Diagnostic Odds Ratio (DOR) were calculated using formulas from Fischer, Bachmann (Fischer, Bachmann, & Jaeschke, 2003). The DOR is a measure of a diagnostic test's overall accuracy (Glas, Lijmer, Prins, Bonsel, & Bossuyt, 2003), and unlike positive and negative predictive values, the DOR does not depend on the prevalence of the disease, facilitating comparisons of tests for meta‐analyses. A DOR value of 20 or more indicates that an instrument has useful screening properties (Fischer et al., 2003).

Cronbach's alpha was calculated to measure internal consistency in the resulting factors of the WURS and ASRS. SPSS version 24.0 was used for the statistical analyses (IBM 26).

## 3 RESULTS

**FIGURE 1:**
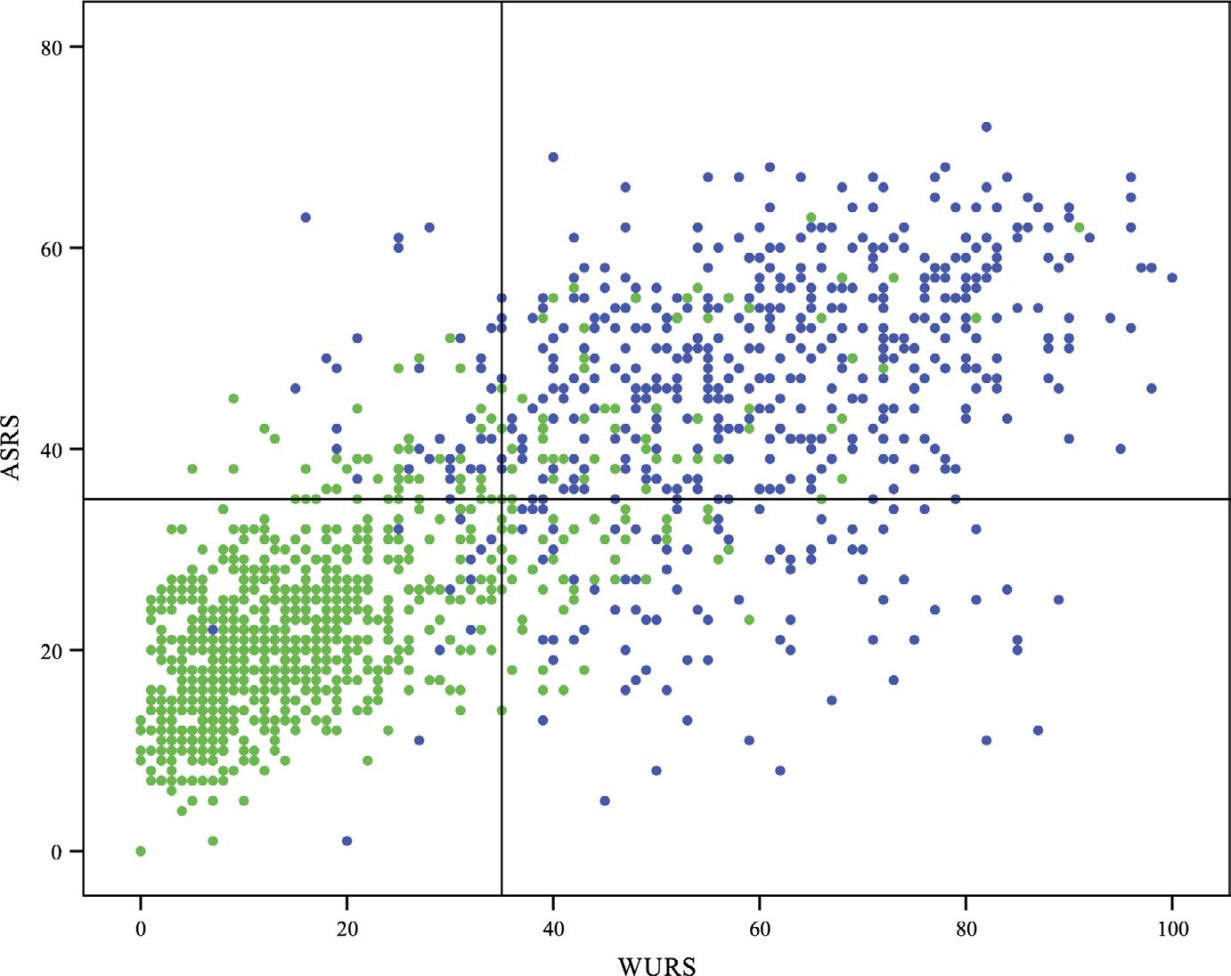
Distribution of WURS and ASRS scores in the ADHD and control samples. Top left: Distribution of ASRS scores. Bottom left: Distribution of WURS scores. Right: Overlap between scores on the WURS and ASRS. Controls are green. ADHD patients are blue. Lines represent a cutoff score of 35; vertical line for WURS, horizontal line for ASRS. This gives a sensitivity of 0.90 and specificity of 0.88 for the WURS, and a sensitivity of 0.80 and specificity of 0.88 for the ASRS

The present study included *n* = 646 clinically assessed adult ADHD patients and *n* = 908 controls, resulting in a total sample of 1,554 participants. The mean ages were 34.0 (*SD *10.3) years in the ADHD group and 29.4 (*SD *7.8) years in the control group (*p* < .01). There were 48.5% females in the ADHD group and 59.9% females in the control group (*p* < .01). The total WURS and ASRS scores were strongly correlated (full sample *r* = .78, *p* < .001; ADHD group *r* = .36, *p* < .001; controls *r* = .70, *p* < .001). Figure 1 shows the distributions of WURS and ASRS scores in the ADHD and control samples, including the correlation between the two. For a subset of patients, we also obtained clinician ratings on whether the patients were currently on (*n* = 420) or off (*n* = 125) pharmacological treatment for ADHD, as well as if they had been treated for ADHD as a child (*n* = 89) or not (*n* = 530). Adults with ADHD on current pharmacological treatment reported a significantly lower ASRS score than the off treatment group, but there was no difference between these groups on the WURS. Adult patients who had been treated for ADHD as a child scored significantly higher on both the ASRS and the WURS compared to those patients who reported no childhood treatment. Mean scores on the ASRS and WURS for the ADHD group and the control group, as well as for the different subgroups within the ADHD group, are shown in Table 1.

**TABLE 1:** Group differences on the ASRS and the WURS

	Controls (*n* = 908)	ADHD (*n* = 646)	Currently on medication (*n* = 420)	Currently off medication (*n* = 125)	No childhood treatment (*n* = 530)	Received childhood treatment (*n* = 89)
ASRS	23.0 (9.8)	45.0 (12.6)**	43.5 (13.3)	48.1 (10.4)**	31.4 (14.4)	41.5 (12.7)*
WURS	17.3 (13.9)	58.2 (17.9)**	58.0 (18.3)	58.6 (17.0)	32.5 (25.2)	55.1 (18.9)**

Note

Mean scores with standard deviations (*SD*) in parentheses. The comparisons were pair wise, with the four comparison groups on the right being subgroups within the adult ADHD group.

* *p* < .01.

** *p* < .001.

### 3.1 Factor analyses

The Principal Component analysis generated a three‐factor solution for the WURS items in the full sample (Table 2) explaining 69.2% of the variance. This solution offered high item loadings on each scale, with a few exceptions. The highest loading items on the first factor were “Temper outbursts, Tantrums” and “Angry”, including items of defiant behavior. The highest loading items on the second factor were “Overall a poor student, Slow learner” and “Trouble with mathematics or numbers,” also including items of inattention. The items with the highest loading on the third factor were “Anxious, Worrying” and “Sad or blue, Depressed, Unhappy.” We thus named the three factors *Aggressiveness and social problems*, *Learning and attention problems* and *Dysthymia*, respectively. Only the item “Unpopular with other children […]” had ambiguous loading with factor loadings below 0.50 on all factors. Internal consistency measured by Cronbach's alpha was 0.967 for the full WURS, and 0.954 for *Aggressiveness and social Problems*, 0.919 for *Learning and attention problems* and 0.897 for *Dysthymia*, respectively.

**TABLE 2:** Rotated factor component matrix for the WURS

As a child I was (or had)	Component		
	1	2	3
Aggressiveness and Social Problems (*30.7% of the variance*)			
WURS 6: Temper outbursts, tantrums	**0.817**	0.204	0.235
WURS 14: Angry	**0.811**	0.172	0.318
WURS 5: Hot‐ or short‐tempered, low boiling point	**0.785**	0.242	0.261
WURS 10: Disobedient with parents, rebellious, sassy	**0.746**	0.214	0.137
WURS 19: Losing control of myself	**0.740**	0.342	0.328
WURS 12: Irritable	**0.738**	0.247	0.401
WURS 8: Stubborn, strong‐willed	**0.673**	0.242	0.475
WURS 13: Moody, ups and downs	**0.672**	0.403	0.336
WURS 20: Tendency to be or act irrational	**0.662**	0.154	0.090
WURS 15: Trouble seeing things from someone else's point of view	**0.652**	0.390	0.115
WURS 22: Trouble with authorities, trouble with school, visits to principal's office	**0.635**	0.374	0.230
WURS 16: Acting without thinking, impulsive	**0.621**	0.559	0.234
WURS 21: Unpopular with other children, did not keep friends for long, did not get along with other children	**0.467**	0.349	0.433
Learning and Attention Problems (*19.5% of the variance*)			
WURS 23: Overall a poor student, slow learner	0.229	**0.783**	0.195
WURS 24: Trouble with mathematics or numbers	0.147	**0.757**	0.209
WURS 25: Not achieving up to potential	0.312	**0.730**	0.370
WURS 1: Concentration problems, easily distracted	0.459	**0.694**	0.310
WURS 7: Trouble with stick‐to‐it‐tiveness, not following through, failing to finish things started	0.369	**0.595**	0.427
WURS 4: Inattentive, daydreaming	0.519	**0.587**	0.330
WURS 17: Tendency to be immature	0.432	**0.542**	0.308
Dysthymia (*19.0% of the variance*)			
WURS 2: Anxious, worrying	0.200	0.240	**0.814**
WURS 9: Sad or blue, depressed, unhappy	0.313	0.151	**0.799**
WURS 11: Low opinion of myself	0.166	0.265	**0.754**
WURS 18: Guilty feelings, regretful	0.230	0.280	**0.728**
WURS 3: Nervous, fidgety	0.334	0.377	**0.684**

Note

PCA with Varimax rotation on the WURS in the full sample. Items sorted by factor loadings. Total variance explained by the three‐factor solution: 69.2%.

A two‐factor solution was generated for the ASRS in the full sample (Table 3), explaining 62.2% of the variance. The first factor included items reflecting symptoms of inattention, the second factor symptoms of hyperactivity and impulsivity. The items reflecting impulsive behavior obtained the highest loadings on the second factor.

**TABLE 3:** Rotated factor component matrix for the ASRS

Circle the number that best describes how you have felt and conducted yourself over the past 6 months	Component	
	1	2
Inattentive (*34.0% of the variance*)		
ASRS5 How often do you have difficulty getting things in order when you have to do a task that requires organization?	0.794	0.263
ASRS2 How often do you have difficulty keeping your attention when you are doing boring or repetitive work?	0.753	0.373
ASRS6 When you have a task that requires a lot of thought, how often do you avoid or delay getting started?	0.739	0.291
ASRS1 How often do you make careless mistakes when you have to work on a boring or difficult project?	0.735	0.327
ASRS4 How often do you have trouble wrapping up the fine details of a project, once the challenging parts have been done?	0.721	0.394
ASRS3 How often do you have difficulty concentrating on what people say to you, even when they are speaking to you directly?	0.679	0.413
ASRS7 How often do you misplace or have difficulty finding things at home or at work?	0.662	0.230
ASRS9 How often do you have problems remembering appointments or obligations?	0.655	0.264
ASRS8 How often are you distracted by activity or noise around you?	0.654	0.437
Hyperactive/Impulsive (*28.2% of the variance*)		
ASRS15 How often do you find yourself talking too much when you are in a social situation?	0.209	0.761
ASRS16 When you're in a conversation, how often do you find yourself finishing the sentences of the people that you are talking to, before they can finish them themselves?	0.236	0.749
ASRS17 How often do you have difficulty waiting your turn in situations when turn‐taking is required?	0.371	0.740
ASRS18 How often do you interrupt others when they are busy?	0.305	0.731
ASRS14 How often do you feel overly active and compelled to do things, like you were driven by a motor?	0.394	0.684
ASRS12 How often do you feel restless or fidgety?	0.579	0.595
ASRS11 How often do you leave your seat in meetings or other situations in which you are expected to remain seated?	0.502	0.577
ASRS13 How often do you have difficulty unwinding and relaxing when you have time to yourself?	0.478	0.574
ASRS10 How often do you fidget or squirm with your hands or your feet when you have to sit down for a long time?	0.514	0.567

Note

PCA with Varimax rotation on the ASRS in the full sample. 62.2% Variance explained in the full sample rotated factor solution. Items sorted by factor loadings.

Internal consistency measured by Cronbach's alpha was 0.952 for the full ASRS score, 0.924 for the Inattentive factor and 0.918 for the Hyperactivity/Impulsivity factor.

### 3.2 The discriminative ability of the WURS and the ASRS

**FIGURE 2:**
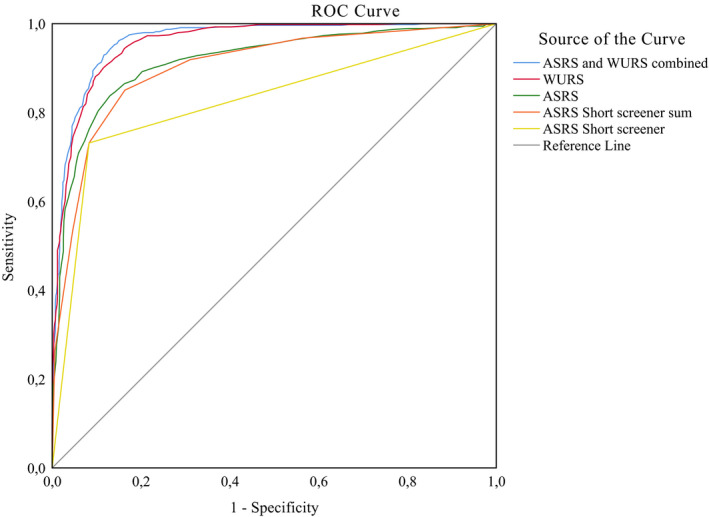
Receiving operator curve illustrating the psychometric properties of the WURS and the ASRS in predicting adult ADHD status. Blue line represents the ASRS and the WURS combined. Red line represents the WURS. Green line represents the ASRS. Orange line represents the ASRS Short screener 6 item sum. Yellow line represents the ASRS Short screener used dichotomously. A steeper curve indicates better discriminatory properties

Figure 2 illustrates the discriminatory values of both the WURS and the ASRS, with an AUC of 0.956 (95% CI: 0.946–0.965) for the WURS, and 0.904 (95% CI: 0.888 ‐ 0.921) for the ASRS. The short screen ASRS had an AUC of 0.903 (95%CI: 0.886–0.920). Combining the two scales gave an AUC of 0.964 (95% CI: 0.955–0.973). There were no significant differences between males and females (data not shown). The optimal cutoff balancing the trade‐off between sensitivity and specificity for the respective scales may vary depending on the aims in the specific clinical or research setting. Table 4 provides cutoff values for 98%, 95%, 90% and 80% sensitivity and specificity, respectively, for both the WURS and the ASRS, including LHs and DORs for each cutoff. Using sum scores from all the factors extracted from PCA, *Learning and attention problems* had the highest AUC of 0.95 (95% CI 0.94–0.96), followed by *Aggressiveness and social problems* with 0.93 (95% CI 0.92–0.94).

**TABLE 4:** Predictive validity of the WURS and the ASRS

Sensitivity
	WURS score	(Specificity)	LH+	LH−	DOR	ASRS score	(Specificity)	LH+	LH−	DOR
0.98	21	(0.71)	3.38	0.03	135.5	16	(0.22)	1.26	0.09	14.6
0.95	29	(0.83)	5.59	0.06	95.6	21	(0.45)	1.73	0.11	18.0
0.90	35	(0.88)	7.50	0.11	64.7	27	(0.71)	3.10	0.14	23.6
0.80	42	(0.93)	11.43	0.22	56.1	35	(0.88)	6.67	0.23	30.8
**Specificity**		**(Sensitivity)**					**(Sensitivity)**			
0.98	56	(0.55)	27.5	0.46	53.4	49	(0.45)	22.5	0.56	34.0
0.95	46	(0.75)	15	0.26	54.5	42	(0.64)	12.8	0.38	32.7
0.90	36	(0.89)	8.9	0.12	63.5	36	(0.79)	7.9	0.23	32.0
0.80	26	(0.97)	4.85	0.04	117.3	30	(0.88)	4.4	0.15	27.8

Note

Complimentary Specificity/Sensitivity given in parenthesis.

Abbreviations: ASRS, Adult ADHD Self‐Report Scale; DOR, diagnostic odds ratio; LH−, Likelihood ratio negative test; LH+, Likelihood ratio positive test; WURS, Wender Utah Rating Scale.

## 4 DISCUSSION

Both the WURS and the ASRS had excellent screening and psychometric properties, with somewhat stronger properties for the WURS. The recommended short screener ASRS performed as well as the full ASRS. A Principal Component analysis confirmed a three‐factor structure of the WURS described in previous studies (Caci et al., 2010; Kouros, Horberg, Ekselius, & Ramklint, 2018; McCann et al., 2000; Stanton & Watson, 2016), albeit with some differences at item level. The well described two‐factor structure was confirmed for the ASRS. Using area under the curve (AUC), our findings fit well with previous cutoff suggestions by Ward et al. (1993) for the WURS and by Kessler et al. (2005) on the full 18 item ASRS (Table 4). The total sum scores on WURS and ASRS were strongly correlated.

The delineation of disorder versus normality is a universal problem when a diagnosis is based on symptoms that are dimensional and normally distributed, and it is of particular concern in a disorder for which controlled stimulant substances with potential for abuse are first‐line treatments (McGough & Barkley, 2004). Thus, establishing validity and accuracy of the two most commonly used screening instruments is vital. Contrary to the critique raised against the WURS for lacking content validity (i.e., diverging from the DSM symptom criteria; Stanton & Watson, 2016), we found a very high criterion validity of WURS (i.e., being highly predictive of an ADHD diagnosis). The items driving this discriminatory ability were part of the *Learning and attention problems* factor of the WURS. The items represent behaviors well recognized as core ADHD symptoms (WURS 1*: Concentration problems, easily distracted* and WURS 7: *Trouble with stick‐to‐it‐tiveness, not following through, failing to finish things started*). Finding that the WURS outperformed the ASRS adds to the ongoing controversy of the defining features of adult ADHD. The factor analysis of the WURS showed that the main factor of the WURS was the *Aggressiveness and social problems*, indicating that these symptoms play an important role in ADHD. Adler et al. (2017) suggested that executive dysfunction is as central as the DSM‐5 symptoms to adult ADHD, while emotional dysregulation has been suggested to be more distinct but nevertheless part of the combined presentation of adult ADHD (Haavik et al., 2010; Shaw, Stringaris, Nigg, & Leibenluft, 2014). In a recent study, both executive function deficits and emotional dyscontrol items have been included as part of expanded versions of screening instruments for adult ADHD, showing the increased focus on these symptoms in recent years (Silverstein et al., 2019). The better discriminatory properties of the WURS are noteworthy as our patients were diagnosed as adults based on a comprehensive clinical evaluation following the ICD/DSM criteria. Thus, even strictly defined adult ADHD patients are more easily distinguished from controls with a broader childhood symptom array than the current DSM core symptoms. This fits well with the well‐established finding that ADHD is characterized by childhood onset and symptoms within domains of executive problems and emotional dysregulation. Although traditionally viewed as comorbid problems, these symptoms rather seem to be characteristic of having ADHD itself. Thus, the broader aspect covered by the WURS may reflect the broader picture that is essentially characteristic of persistent ADHD.

Although the AUC was only slightly better for the WURS than for the ASRS, the differences in the diagnostic odds ratios were considerable, as the WURS had an overall better specificity with intact sensitivity. Our findings suggest the ASRS is not adequate in situations requiring very high sensitivity, as the specificity was merely 0.45 at sensitivity 0.95.

The retrospective focus of the WURS evoking a developmental frame and spanning over a longer period of time may furthermore elicit responses that separate better between adult patients with ADHD and controls. Another possible explanation for the better screening properties of the WURS could be that some of the patients have ADHD in partial remission (and thus a low ASRS score). We found that adult ADHD patients on current pharmacological (mainly stimulant) treatment reported less current symptoms of ADHD on the ASRS compared to those who were not on medication, but there were no statistical differences on the WURS. Furthermore, patients treated for ADHD in childhood reported more symptoms than those who had not been treated in childhood on both the WURS and the ASRS, indicating a more severe and persistent phenotype (Halmoy et al., 2009).

### 4.1 Strengths and limitations

The present findings should be viewed in light of some limitations. There are problems related to the use of self‐report measures because measures that employ a retrospective approach might be affected by memory biases and lack of recall. McGough and Barkley (2004) argued that “a major obstacle to retrospective diagnosis is that it is significantly biased by current functioning.” However, our findings show that the retrospective WURS did better than reports of current ADHD symptoms in differentiating adult ADHD patients from controls. This is in line with previous studies on the WURS by, for example, Fossati et al. (2001) showing excellent short‐term retest reliability. Both Fossati et al. (2001) and Grogan and Bramham (2016) found that current mood symptoms do not affect the accuracy of retrospective self‐ratings of childhood ADHD symptoms. A recent study has found that the WURS even has acceptable retest reliability over the time span of several years (Lundervold, Vartiainen, Jensen, & Haavik, 2019). The ASRS on the other hand may be more affected by short‐term confounders such as affective fluctuations (Lundervold et al., 2011), time of day (Franke et al., 2012) and sleep problems (Benjamins et al., 2016; Brevik et al., 2017). Comorbid psychiatric disorders could have influenced findings, but to maintain external validity we chose not to control for these, as ADHD is more often comorbid than not (Singh, 2008; Sobanski, 2006).

This study was based on an adult ADHD sample ascertained in adulthood, meaning that it is uncertain whether the patients included would have obtained a childhood diagnosis of ADHD, with the expected symptomatic trajectory. This is potentially an important caveat, as some recent studies have put into question ADHD as a neurodevelopmental disorder, highlighting both discontinuation of childhood symptoms as well as a possible adult onset ADHD phenotype (Agnew‐Blais et al., 2016; Caye et al., 2016; Moffitt et al., 2015).

We used a clinically validated patient sample and a representative population control sample, which strengthens the clinical utility of our findings. Our control sample was randomly recruited from the Norwegian Medical Birth Registry, without any formal exclusion criteria, so there is a potential for some undiagnosed cases of ADHD in the control group. However, screening instruments are generally more useful in at risk populations rather than in the general population, where the performance of the screening tools could be overstated.

## 5 CONCLUSION

The Norwegian translation of both the ASRS and the WURS had excellent psychometric properties and can be used independently for screening and diagnostic assessment for ADHD. We found that the WURS had even better screening properties than the ASRS, in spite of our sample being clinically assessed and diagnosed in adulthood. The wider WURS dimensions of aggression, learning problems and emotional lability were highly relevant to identify adult ADHD in our sample, supporting a broader conceptualization of ADHD. With their different temporal focus and clinically relevant symptom domains, we recommend using the ASRS and the WURS jointly to assess for adult ADHD.

## CONFLICT OF INTEREST

JH has received lecture honoraria as part of continuing medical education programs sponsored by Novartis, Eli Lilly and Company, and Janssen‐Cilag. The other authors report no potential conflicts of interest.

## AUTHOR CONTRIBUTION

All authors were involved in the conception and design of the study. JH supervised the data collection for the study. All authors were involved in the data analysis and interpretation, drafting the article and critical revision of the article. All authors approved of the final version to be published.

## Data Availability

The data that support the findings of this study are available from the corresponding author upon reasonable request.

## References

[brb31605-bib-0001] Adler, L. A. , Faraone, S. V. , Spencer, T. J. , Berglund, P. , Alperin, S. , & Kessler, R. C. (2017). The structure of adult ADHD. International Journal of Methods in Psychiatric Research, 26(1), e1555. 10.1002/mpr.1555 PMC5405726 28211596

[brb31605-bib-0002] Adler, L. A. , Spencer, T. , Faraone, S. V. , Kessler, R. C. , Howes, M. J. , Biederman, J. , & Secnik, K. (2006). Validity of pilot Adult ADHD Self‐ Report Scale (ASRS) to Rate Adult ADHD symptoms. Annals of Clinical Psychiatry, 18, 145–148. 16923651 10.1080/10401230600801077

[brb31605-bib-0003] Agnew‐Blais, J. C. , Polanczyk, G. V. , Danese, A. , Wertz, J. , Moffitt, T. E. , & Arseneault, L. (2016). Evaluation of the persistence, remission, and emergence of attention‐deficit/hyperactivity disorder in young adulthood. JAMA Psychiatry, 73, 713–720. 27192174 10.1001/jamapsychiatry.2016.0465 PMC5475268

[brb31605-bib-0004] American Psychiatric Association (2000). Diagnostic and statistical manual of mental disorders. Washington, DC: American Psychiatric Association.

[brb31605-bib-0005] American Psychiatric Association (2013). Diagnostic and statistical manual of mental disorders (5th ed.). Washington, DC: American Psychiatric Association.

[brb31605-bib-0006] Benjamins, J. S. , Migliorati, F. , Dekker, K. , Wassing, R. , Moens, S. , Blanken, T. F. , … Van Someren, E. J. W. (2016). Insomnia heterogeneity: Characteristics to consider for data‐driven multivariate subtyping. Sleep Medicine Reviews, 36, 71–81. 29066053 10.1016/j.smrv.2016.10.005

[brb31605-bib-0007] Brevik, E. J. , Lundervold, A. J. , Halmoy, A. , Posserud, M.‐B. , Instanes, J. T. , Bjorvatn, B. , & Haavik, J. (2017). Prevalence and clinical correlates of insomnia in adults with attention‐deficit hyperactivity disorder. Acta Psychiatrica Scandinavica, 136(2), 220–227. 28547881 10.1111/acps.12756

[brb31605-bib-0008] Caci, H. M. , Bouchez, J. , & Baylé, F. J. (2010). An aid for diagnosing attention‐deficit/hyperactivity disorder at adulthood: Psychometric properties of the French versions of two Wender Utah Rating Scales (WURS‐25 and WURS‐K). Comprehensive Psychiatry, 51, 325–331. 20399344 10.1016/j.comppsych.2009.05.006

[brb31605-bib-0009] Caye, A. , Rocha, T. , Anselmi, L. , Murray, J. , Menezes, A. M. B. , Barros, F. C. , … Rohde, L. A. (2016). Attention‐deficit/hyperactivity disorder trajectories from childhood to young adulthood: Evidence from a birth cohort supporting a late‐onset syndrome. JAMA Psychiatry, 73, 705–712. 27192050 10.1001/jamapsychiatry.2016.0383

[brb31605-bib-0010] Faraone, S. V. , Asherson, P. , Banaschewski, T. , Biederman, J. , Buitelaar, J. K. , Ramos‐Quiroga, J. A. , … Franke, B. (2015). Attention‐deficit/hyperactivity disorder. Nature Reviews Disease Primers, 1, 15020. 10.1038/nrdp.2015.20 27189265

[brb31605-bib-0011] Fayyad, J. , Sampson, N. A. , Hwang, I. , Adamowski, T. , Aguilar‐Gaxiola, S. , Al‐Hamzawi, A. , … Kessler, R. C. (2017). The descriptive epidemiology of DSM‐IV Adult ADHD in the World Health Organization World Mental Health Surveys. Attention Deficit and Hyperactivity Disorders, 9, 47–65. 27866355 10.1007/s12402-016-0208-3 PMC5325787

[brb31605-bib-0012] Field, A. (2013). Discovering statistics using IBM SPSS statistics. Sage.

[brb31605-bib-0013] Fischer, J. E. , Bachmann, L. M. , & Jaeschke, R. (2003). A readers' guide to the interpretation of diagnostic test properties: Clinical example of sepsis. Intensive Care Medicine, 29, 1043–1051. 12734652 10.1007/s00134-003-1761-8

[brb31605-bib-0014] Fossati, A. , di Ceglie, A. , Acquarini, E. , Donati, D. , Donini, M. , Novella, L. , & Maffei, C. (2001). The retrospective assessment of childhood attention deficit hyperactivity disorder in adults: Reliability and validity of the Italian version of the Wender Utah Rating Scale. Comprehensive Psychiatry, 42, 326–336. 11458308 10.1053/comp.2001.24584

[brb31605-bib-0015] Franke, B. , Faraone, S. V. , Asherson, P. , Buitelaar, J. , Bau, C. H. D. , Ramos‐Quiroga, J. A. , … Reif, A. (2012). The genetics of attention deficit/hyperactivity disorder in adults, a review. Molecular Psychiatry, 17, 960–987. 22105624 10.1038/mp.2011.138 PMC3449233

[brb31605-bib-0016] Gisbert, L. , Richarte, V. , Corrales, M. , Ibáñez, P. , Bosch, R. , Casas, M. , & Ramos‐Quiroga, J. A. (2018). The impact of emotional lability symptoms during childhood in adults with ADHD. Journal of Attention Disorders, 22, 581–590. 28760090 10.1177/1087054717719534

[brb31605-bib-0017] Glas, A. S. , Lijmer, J. G. , Prins, M. H. , Bonsel, G. J. , & Bossuyt, P. M. (2003). The diagnostic odds ratio: A single indicator of test performance. Journal of Clinical Epidemiology, 56, 1129–1135. 14615004 10.1016/s0895-4356(03)00177-x

[brb31605-bib-0018] Grogan, K. , & Bramham, J. (2016). Current mood symptoms do not affect the accuracy of retrospective self‐ratings of childhood ADHD symptoms. Journal of Attention Disorders, 20, 1039–1046. 24691528 10.1177/1087054714528536

[brb31605-bib-0019] Haavik, J. , Halmoy, A. , Lundervold, A. J. , & Fasmer, O. B. (2010). Clinical assessment and diagnosis of adults with attention‐deficit/hyperactivity disorder. Expert Review of Neurotherapeutics, 10, 1569–1580. 20925472 10.1586/ern.10.149

[brb31605-bib-0020] Halmoy, A. , Fasmer, O. B. , Gillberg, C. , & Haavik, J. (2009). Occupational outcome in adult ADHD: Impact of symptom profile, comorbid psychiatric problems, and treatment: A cross‐sectional study of 414 clinically diagnosed adult ADHD patients. Journal of Attention Disorders, 13, 175–187. 19372500 10.1177/1087054708329777

[brb31605-bib-0021] Halmoy, A. , Halleland, H. , Dramsdahl, M. , Bergsholm, P. , Fasmer, O. B. , & Haavik, J. (2010). Bipolar symptoms in adult attention‐deficit/hyperactivity disorder: A cross‐sectional study of 510 clinically diagnosed patients and 417 population‐based controls. Journal of Clinical Psychiatry, 71, 48–57. 20129005 10.4088/JCP.08m04722ora

[brb31605-bib-0022] IBM (2016). IBM SPSS Statistics for Macintosh Statistics for Macintosh, Version 24.0. Armonk, NY: IBM Corp.

[brb31605-bib-0023] Katzman, M. A. , Bilkey, T. S. , Chokka, P. R. , Fallu, A. , & Klassen, L. J. (2017). Adult ADHD and comorbid disorders: Clinical implications of a dimensional approach. BMC Psychiatry, 17, 302. 28830387 10.1186/s12888-017-1463-3 PMC5567978

[brb31605-bib-0024] Kessler, R. C. , Adler, L. A. , Ames, M. , Demler, O. , Faraone, S. , Hiripi, E. , … Walters, E. (2005). The World Health Organization adult ADHD self‐report scale (ASRS): A short screening scale for use in the general population. Psychological Medicine, 35, 245–256. 15841682 10.1017/s0033291704002892

[brb31605-bib-0025] Kessler, R. C. , Adler, L. A. , Gruber, M. J. , Sarawate, C. A. , Spencer, T. , & van Brunt, D. L. (2007). Validity of the World Health Organization Adult ADHD Self‐Report Scale (ASRS) Screener in a representative sample of health plan members. International Journal of Methods in Psychiatric Research, 16, 52–65. 17623385 10.1002/mpr.208 PMC2044504

[brb31605-bib-0026] Kouros, I. , Horberg, N. , Ekselius, L. , & Ramklint, M. (2018). Wender Utah Rating Scale‐25 (WURS‐25): Psychometric properties and diagnostic accuracy of the Swedish translation. Upsala Journal of Medical Sciences, 123, 230–236. 30373435 10.1080/03009734.2018.1515797 PMC6327570

[brb31605-bib-0027] Lundervold, A. J. , Adolfsdottir, S. , Halleland, H. , Halmøy, A. , Plessen, K. , & Haavik, J. (2011). Attention Network Test in adults with ADHD – The impact of affective fluctuations. Behavioral and Brain Functions, 7, 27. 21794128 10.1186/1744-9081-7-27 PMC3168400

[brb31605-bib-0028] Lundervold, A. J. , Vartiainen, H. , Jensen, D. , & Haavik, J. (2019). Test‐retest reliability of the 25‐item version of Wender Utah Rating Scale. Impact of current ADHD severity on retrospectively assessed childhood symptoms. Journal of Attention Disorders, 1087054719879501. Epub ahead of print 10.1177/1087054719879501 31583933

[brb31605-bib-0029] McCann, B. S. , Scheele, L. , Ward, N. , & Roy‐Byrne, P. (2000). Discriminant validity of the Wender Utah Rating Scale for attention‐deficit/hyperactivity disorder in adults. The Journal of Neuropsychiatry and Clinical Neurosciences, 12(2), 240–245. 11001603 10.1176/jnp.12.2.240

[brb31605-bib-0030] McGough, J. J. , & Barkley, R. A. (2004). Diagnostic controversies in adult attention deficit hyperactivity disorder. The American Journal of Psychiatry, 161, 1948–1956. 15514392 10.1176/appi.ajp.161.11.1948

[brb31605-bib-0031] Moffitt, T. E. , Houts, R. , & Asherson, P. , Belsky, D. W. , Corcoran, D. L. , Hammerle, M. , … Caspi, A. (2015). Is adult ADHD a childhood‐onset neurodevelopmental disorder? Evidence from a four‐decade longitudinal cohort study. The American Journal of Psychiatry, 172, 967–977. 25998281 10.1176/appi.ajp.2015.14101266 PMC4591104

[brb31605-bib-0032] Polanczyk, G. , de Lima, M. S. , Horta, B. L. , Biederman, J. , & Rohde, L. A. (2007). The worldwide prevalence of ADHD: a systematic review and metaregression analysis. The American Journal of Psychiatry, 164(6), 942–948. 17541055 10.1176/ajp.2007.164.6.942

[brb31605-bib-0033] Shaw, P. , Stringaris, A. , Nigg, J. , & Leibenluft, E. (2014). Emotion dysregulation in attention deficit hyperactivity disorder. The American Journal of Psychiatry, 171, 276–293. 24480998 10.1176/appi.ajp.2013.13070966 PMC4282137

[brb31605-bib-0034] Silverstein, M. J. , Faraone, S. V. , Alperin, S. , Leon, T. L. , Biederman, J. , Spencer, T. J. , & Adler, L. A. (2019). Validation of the expanded versions of the adult ADHD self‐report Scale v1.1 Symptom Checklist and the Adult ADHD Investigator Symptom Rating Scale. Journal of Attention Disorders, 23, 1101–1110. 29419345 10.1177/1087054718756198

[brb31605-bib-0035] Singh, I. (2008). Beyond polemics: Science and ethics of ADHD. Nature Reviews Neuroscience, 9, 957–964. 19020513 10.1038/nrn2514

[brb31605-bib-0036] Sobanski, E. (2006). Psychiatric comorbidity in adults with attention‐deficit/hyperactivity disorder (ADHD). European Archives of Psychiatry and Clinical Neuroscience, 256(Suppl. 1), i26–i31. 16977548 10.1007/s00406-006-1004-4

[brb31605-bib-0037] Stanton, K. , & Watson, D. (2016). An examination of the structure and construct validity of the Wender Utah Rating Scale. Journal of Personality Assessment, 98(5), 545–552. 27050760 10.1080/00223891.2016.1152579

[brb31605-bib-0038] Turgay, A. , Goodman, D. W. , Asherson, P. , Lasser, R. A. , Babcock, T. F. , Pucci, M. L. , & Barkley, R. (2012). Lifespan persistence of ADHD: The life transition model and its application. Journal of Clinical Psychiatry, 73, 192–201. 22313720 10.4088/JCP.10m06628

[brb31605-bib-0039] Ustun, B. , Adler, L. A. , Rudin, C. , Faraone, S. V. , Spencer, T. J. , Berglund, P. , … Kessler, R. C. (2017). The World Health Organization Adult Attention‐Deficit/Hyperactivity Disorder Self‐Report Screening Scale for DSM‐5. JAMA Psychiatry, 74, 520–526. 28384801 10.1001/jamapsychiatry.2017.0298 PMC5470397

[brb31605-bib-0040] van de Glind, G. , van den Brink, W. , Koeter, M. W. J. , Carpentier, P.‐J. , van Emmerik‐van Oortmerssen, K. , Kaye, S. , … Levin, F. R. (2013). Validity of the Adult ADHD Self‐Report Scale (ASRS) as a screener for adult ADHD in treatment seeking substance use disorder patients. Drug and Alcohol Dependence, 132, 587–596. 23660242 10.1016/j.drugalcdep.2013.04.010 PMC4083506

[brb31605-bib-0041] Ward, M. F. , Wender, P. H. , & Reimherr, F. W. (1993). The Wender Utah Rating Scale: An aid in the retrospective diagnosis of childhood attention deficit hyperactivity disorder. The American Journal of Psychiatry, 150, 885–890. 8494063 10.1176/ajp.150.6.885

[brb31605-bib-0042] Wender, P. H. (1995). Attention‐deficit hyperactivity disorder in adults. New York, NY: Oxford University Press.

[brb31605-bib-0043] WHO (1992). The ICD‐10 classification of mental and behavioural disorders: Clinical descriptions and diagnostic guidelines. Geneva, Switzerland: World Health Organization.

[brb31605-bib-0044] Zohar, A. H. , & Konfortes, H. (2010). Diagnosing ADHD in Israeli adults: The psychometric properties of the adult ADHD Self Report Scale (ASRS) in Hebrew. The Israel Journal of Psychiatry and Related Sciences, 47, 308–315. 21270505

