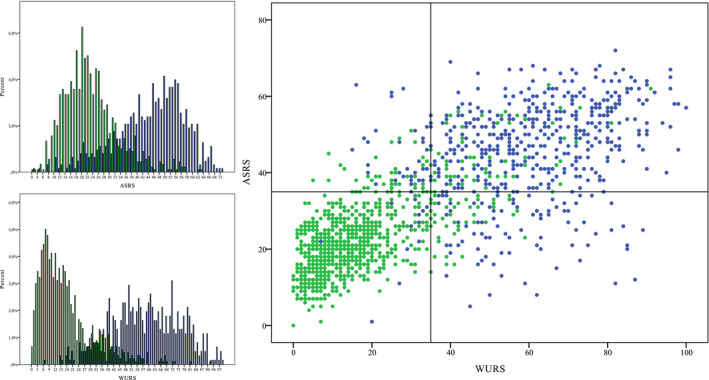# Validity and accuracy of the Adult Attention‐Deficit/Hyperactivity Disorder (ADHD) Self‐Report Scale (ASRS) and the Wender Utah Rating Scale (WURS) symptom checklists in discriminating between adults with and without ADHD

**DOI:** 10.1002/brb3.2067

**Published:** 2021-03-19

**Authors:** Erlend Joramo Brevik, Astri J. Lundervold, Jan Haavik, Maj‐Britt Posserud

**Affiliations:** ^1^ Division of Psychiatry Haukeland University Hospital Bergen Norway; ^2^ Department of Biomedicine K.G. Jebsen Centre for Neuropsychiatric Disorders University of Bergen Bergen Norway; ^3^ Department of Biological and Medical Psychology University of Bergen Bergen Norway; ^4^ Department of Clinical Medicine University of Bergen Bergen Norway

The article by Brevik et al. (2020) was published with incorrect figure 1. The correct figure is placed below.

The author apologizes for the error.